# Digestion-Free Middle-Down Mass Spectrometry Method for Absolute Quantification of Conjugated Payload from Antibody-Drug Conjugates

**DOI:** 10.1021/acs.analchem.4c03383

**Published:** 2024-09-19

**Authors:** Jiaqi Yuan, Hui Yin Tan, Yue Huang, Anton I. Rosenbaum

**Affiliations:** Integrated Bioanalysis, Clinical Pharmacology & Safety Sciences, R&D, AstraZeneca, South San Francisco, California 94080, United States

## Abstract

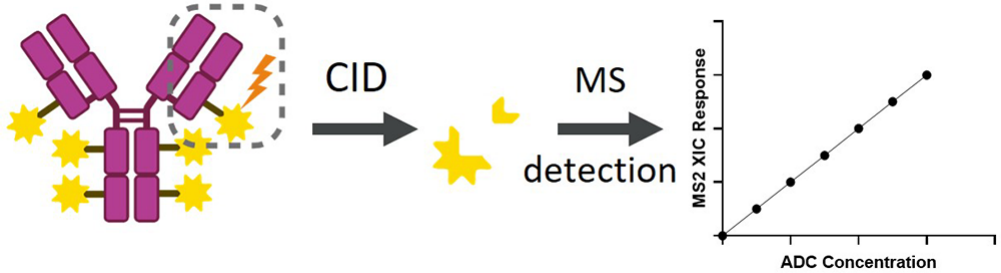

Antibody-drug conjugate (ADC) is a therapeutic modality that aims to improve payload delivery specificity and reduce systemic toxicity. Considering the complex structure of ADCs, various bioanalytical methods by liquid chromatography coupled with mass spectrometry (LC-MS), ligand binding assay (LBA) and hybrid LBA-LC-MS approaches have been established for ADC characterization and quantification. LCMS-based assays enable drug-antibody ratio (DAR) sensitive quantification of the conjugated payload. Typically, for quantitative, DAR-sensitive, assessment by LC-MS/MS,the conjugated payload is enzymatically liberated and quantified. Despite recent advances in ADC bioanalytical methods, the DAR-sensitive quantification of noncleavable linker ADCs by LC-MS/MS remains challenging. Thus, we developed a novel digestion-free middle-down mass spectrometry (DF-MDMS) using a collision-induced dissociation approach for absolute quantification of conjugated payload from four different ADCs in a biological matrix with minimum sample preparation. These results demonstrate that ADCs with different linker-payload structures can be quantified, including a noncleavable linker ADC, trastuzumab emtansine. It also shows that the assay sensitivity is comparable to the conventional ADC quantification method by linker-payload cleavage using enzyme, while the assay dynamic range depends on factors including payload ionization and dissociation efficiency, DAR and its distribution, and species abundance. By demonstrating absolute quantification of both cleavable and noncleavable linker ADCs, this novel middle-down ADC approach demonstrates its potential application in bioanalysis and analytical characterization, especially for early discovery where high-throughput screening is required as the new approach saves time and resources by not requiring enzymatic digestion for cleavable ADCs or development of anti-payload antibodies for noncleavable linker ADCs.

## Introduction

Over the past decades, antibody-drug conjugates (ADCs) have become an increasingly important therapeutic modality in the biopharmaceutical industry. Currently, there are 13 approved ADCs, and many more under evaluation in clinical trials.^1^ ADCs are designed to improve therapeutic index (TI) by targeted payload delivery to increase efficacy and limit the payload off-target toxicity.^2^ ADCs consist of antibody, linker, and payload and can be designed to carry various payloads to different targets and release the payload through a plethora of mechanisms.^3^ Due to the structural complexity of ADCs, multiple bioanalytical assays can be applied to ADC characterization and the understanding of ADC pharmacokinetic properties and payload deconjugation rate, including total antibody, unconjugated payload, and ADC, the latter using either a ligand binding assay (LBA) or liquid chromatography-mass spectrometry (LC-MS)-based assay (Table 1).^4^ While an LBA method requires anti-payload for intact ADC quantification, an LCMS-based method can bypass this limitation. Furthermore, for drug-antibody ratio (DAR)-sensitive quantification of ADC concentration, LBA methods require that capture and detection efficiency be DAR dependent, whereas LCMS-based methods actually require the converse assumption to ensure accurate DAR quantification.

**Table 1:** Overview of ADC Assay Bioanalytical Methods^a^

ADC assay	LBA-based	Mass spectrometry-based		
		Intact	DF-MDMS	MRM
DAR information	Case-by-case	Direct quantification	Indirect quantification	Indirect quantification
Capture reagent	Anti-payload	Anti-Fc/Anti-Idiotype	Anti-Fc/Anti-Idiotype	Anti-Fc/Anti-Idiotype
Enzymatic cleavage	No	No	No	Yes
Relative sensitivity	Medium-high	Low-medium	Medium-high	Medium-high
Cost	Low-medium	Medium-high	Low-medium	Low-medium
Sample prep time	Fast	Fast	Fast	Slow
Data acquisition and processing time	Fast	Slow-moderate	Moderate	Moderate
Limitation	• Capture and detection antibody binding efficiency might not be DAR sensitive	• Assumes all DAR species have similar ionization efficiency	• Requires payload-specific product ion for quantification	• Assumes all DAR species have similar capture and digestion efficiency
	• Highly dependent on critical reagent generation that requires long lead times, substantial costs and continued availability	• Requires expensive instrumentation and specialized expertise	• Assumes all DAR species have similar ionization efficiency and dissociation efficiency	• Provides average DAR
				• Works only for cleavable linkers

a All methods listed would require reference materials of every DAR species to establish a calibration curve for true absolute quantification of each DAR species in particular for high DAR molecules and rapidly-deconjugating ADCs.

Despite the various approaches currently available for ADC quantification, LCMS-based bioanalytical challenges still remain, especially for ADCs with linkers that cannot be enzymatically cleaved. In such cases, conjugated payload from ADCs can be analyzed at intact or subunit levels by LC-HRMS.^5−7^ However, it is typically limited by low sensitivity and low throughput due to the complex and time-consuming data deconvolution process, which could be burdensome to support the bioanalysis of large studies. Furthermore, the conventional enzyme release of the payload approach may not be suitable for the analysis of ADC biotransformation due to structural changes of payload or linker.^8^ Besides that, payload may be released from noncleavable linker chemically and measured by LC-MS.^9^ However, it depends on the chemical structure of the linker-payload and requires a bespoke methodology that cannot be directly applied to other cases.

Here, we report a digestion-free middle-down mass spectrometry (DF-MDMS) method for the absolute quantification of several ADCs on a ZenoTOF 7600 mass spectrometer (SCIEX). Collision-Induced Dissociation (CID) was used for absolute quantification of the ADC-conjugated payload. We applied immunocapture to extract ADCs from biological matrices, followed by acid elution from beads and optional reduction and deglycosylation. The results demonstrate that both cleavable and noncleavable linker ADCs can be quantified using this DF-MDMS method with sufficient sensitivity. This method can streamline ADC quantification, especially in the case of high-throughput analysis of noncleavable linker ADCs.

## Experimental Section

### Materials and Reagents

ADC2 DAR4, ADC2 DAR8, and trastuzumab emtansine stock solutions were provided by AstraZeneca (London, UK). ADC1 stock solution was provided by AstraZeneca (Gaithersburg, MD). Mouse and cynomolgus monkey plasma were purchased from BioIVT. Biotinylated antihuman Fc capture antibody (A80-304B) was purchased from Bethyl Laboratories. SMART IA streptavidin magnetic beads, tris(hydroxymethyl)aminomethane (Tris) buffer (pH 7.5), formic acid (FA), trifluoroacetic acid (TFA) and dithiothreitol (DTT) were purchased from Thermo Fisher Scientific. EndoS was purchased from New England Biolabs. 1 M Tris-HCl, pH 9.5, was purchased from Teknova.

### Sample Preparation and Processing

Standards and Quality Controls (QCs) were prepared by spiking ADC into mouse/cynomolgus monkey plasma. Then, the standards and QCs were incubated with 40 μL of SMART IA streptavidin magnetic beads conjugated with biotinylated antihuman Fc (4 μg) for 2 h at ambient temperature, 1200 rpm to enrich the ADC from plasma. After the enrichment step, the supernatant was removed, and the beads were washed extensively twice with 50 mM Tris, pH 7.5, followed by twice with water (350 μL each wash). The ADC was then eluted off of the beads with 0.1% (ADC2 DAR4 and trastuzumab emtansine) or 1% FA (ADC2 DAR8 and ADC1). For ADC2 DAR8 and ADC1, the eluates were directly injected onto LC-HRMS. For ADC2 DAR4 and trastuzumab emtansine, the eluates were neutralized with 0.2 M Tris-HCl, pH 9.5, and reduced with DTT (2 h at 37 °C). Finally, the ADCs were deglycosylated with EndoS by incubation at 37 °C for 1 h, before LC-HRMS analysis.

### Data Acquisition on LC-HRMS

The chromatography was performed on a Thermo Fisher Scientific MabPac column (PN088648) using SCIEX Exion LC (0.5 mL/min and 80 °C) with 0.1% FA in water/acetonitrile as mobile phases (25–45% B as gradient) for ADC2 DAR4 and trastuzumab emtansine or a Waters BioResolve RP mAb polyphenyl column (PN186009017) with 1% FA and 0.01% TFA in water/ACN as mobile phases (26–40% B as gradient) for ADC2 DAR8 and ADC1. Mass spectrometry was performed using a SCIEX 7600 Zeno TOF system. Table S1 shows the mass spectrometric parameters.

### Data Processing and Quantification

The relevant *m*/*z* of light or heavy chain with a different number of conjugated drugs was isolated as precursor, followed by CID. For quantification, the payload-specific product ion peak was integrated with SCIEX Analytics (v3.1.5.3945) software with automatic peak integration (AutoPeak/MQ4) with 0.02 Da as the extraction range. A linear least-squares regression (1/concentration^2^ weighted) algorithm was used to fit the peak area of MS ion(s) versus concentration. Accuracy of standards and QCs was calculated by comparing the back-calculated value from the calibration curve with the theoretical concentration. Table S2 shows the data processing parameters.

## Results and Discussion

**Figure 1:**
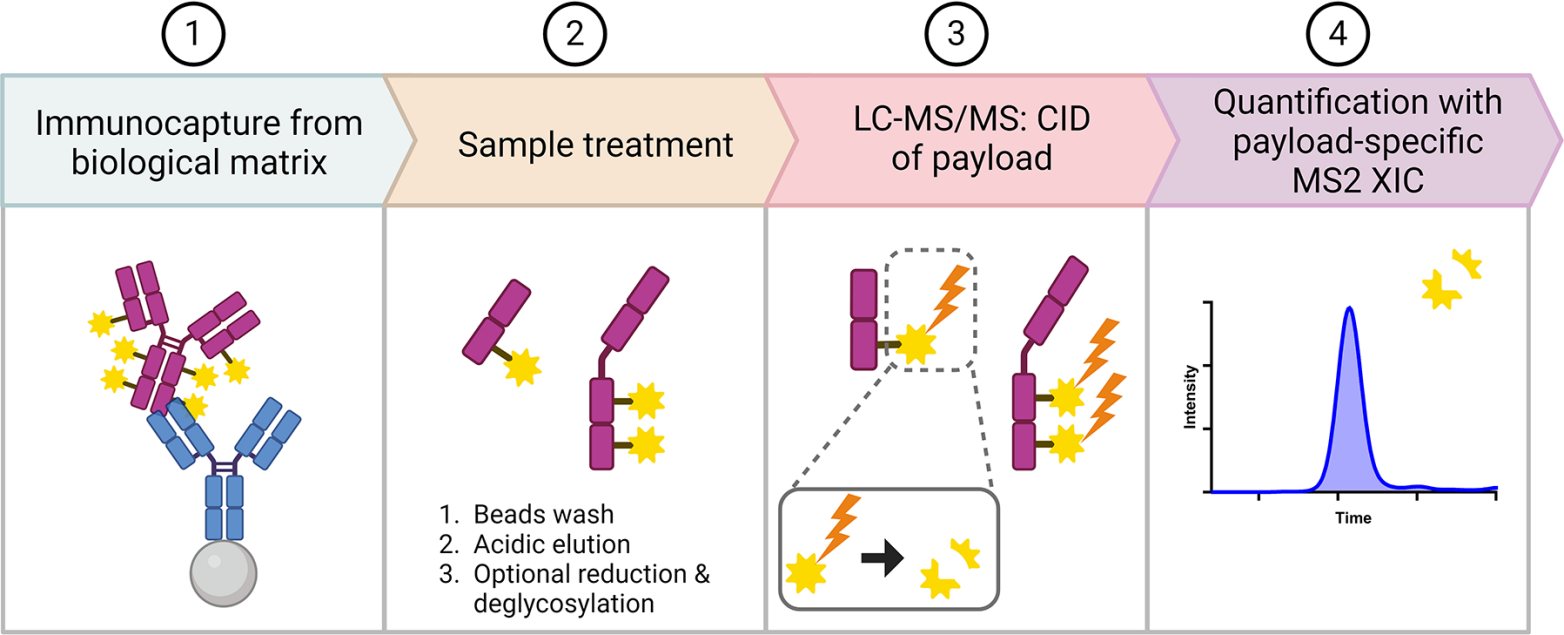
Overall workflow of the DF-MDMS quantification approach. This method utilizes CID of the conjugated payload from ADC. Created with BioRender.com.

In contrast to a conventional LCMS-based bioanalytical method to measure ADC with a cleavable linker that requires enzymatic digestion to release the payload, DF-MDMS quantification uses CID to quantify the payload from ADC without enzymatic digestion. The overall workflow is shown in Figure 1. ADCs were immunocaptured by magnetic beads conjugated with capture antibody and eluted with acid, followed by optional reduction if interchain disulfide bonds exist and/or deglycosylation when needed, especially for heterogeneous ADCs whose spectra can be complicated further by their glycosylation profiles. This further breaks down the intact ADC into single chains, which could be separated chromatographically, reducing complexity and enabling higher resolution for quantification. The extracts were injected into LC coupled with the ZenoTOF 7600 system. Optimized collision energy was applied to dissociate the payload, which permits ADC quantification using signature payload MS^2^ product ions.

To demonstrate the application of the DF-MDMS quantification method, four different ADCs in biological matrices were examined: three cleavable linker ADCs with TOP1i payload and one noncleavable linker ADC, trastuzumab emtansine.^10^ The parameters and assay results of each ADC are summarized in Table 2, including molecule and sample preparation details, data acquisition parameters, assay dynamic range, etc. The overall results show that assays successfully met accuracy acceptance criteria at ±20%/25% for LLOQ; ≥75% of standards and ≥66.7% of QCs met acceptance criteria. Correlation coefficient (*r*) for standard curves was ≥0.985. The assay setup and parameter optimization are discussed in the following sections.

**Table 2:** Summary of ADC Quantification Examples Using the DF-MDMS Absolute Quantification Method^a^

ADC name	ADC1		ADC2 (DAR8)		ADC2 (DAR4)		trastuzumab emtansine	
Antibody Isotype	IgG1		IgG1		IgG1		IgG1	
Payload	TOP1i (AZ14170132)		TOP1i (AZ14170132)		TOP1i (AZ14170132)		Maytansine Derivative (DM-1)	
Linker	Val-Ala-PEG8 (Cleavable)		Val-Ala-PEG8 (Cleavable)		Val-Ala-PEG8 (Cleavable)		SMCC (Non-Cleavable)	
Conjugation Site	Cysteine		Cysteine		Cysteine		Lysine	
DAR	8		8		4		3.5	
Matrix	CD1 Mouse Plasma		Cynomolgus Monkey Plasma		CD1 Mouse Plasma		CD1 Mouse Plasma	
Reduction and Deglycosylation	No		No		Yes		Yes	
Monitored Species	LC1	HC3	LC1	HC3	LC1	HC2	LC1	LC2
MS1 Resolution	Open		Open		Low		Low	
Precursor Ion (*m*/*z*)	1800		1800		1375.3^18+^	1409.5^37+^	1356^18+^, 1436^17+^	1409.6^18+^
Product Ion Used for Quantification (*m*/*z*)	360.2^+^, 404.2^+^, 431.2^+^, 475.2^+^		360.2^+^, 404.2^+^, 431.2^+^, 475.2^+^		431.2^+^	475.2^+^	547.2^+^	
Collision Energy (eV)	100		120		120		60	
Assay Calibration Range (ng/mL)	200–7500	50–7500	100–7500	50–7500	1800–45000	2700–45000	3000–90000	9000–90000
Standard Curve Correlation Coefficient (*r*)	0.997	0.998	0.993	0.990	0.988	0.988	0.988	0.986
Replicates of Standards (*n*)	12	16	14	16	18	16	18	14
Passing Percentage of Standards (%)	91.7	100.0	78.6	75.0	83.3	100.0	88.9	78.6
%CV of Standards	8.02	5.03	10.08	10.66	10.37	10.67	10.85	8.36
Replicates of QCs (*n*)	18	30	16	20	36	30	24	18
Passing Percentage of QCs (%)	94.4	96.7	81.3	90.0	75.0	86.7	83.3	77.8
%CV of QCs	8.07	10.68	11.74	14.20	10.18	10.24	7.46	6.82

a Acceptance criteria were set at ±20%/25% (LLOQ) for accuracy; ≥75% of standards and ≥66.7% of QCs meet acceptance criteria and correlation coefficient (*r*) for standard curves ≥0.985. LC1, light chain with 1 payload; LC2, light chain with 2 payloads; HC2, heavy chain with 2 payloads; HC3, heavy chain with 3 payloads; CV-coefficient of variation.

Reduction and deglycosylation are optional procedures in the DF-MDMS quantification workflow. Among four ADCs, ADC1 and ADC2 DAR8 are stochastic cysteine-conjugated ADCs, which means they do not have interchain disulfide bonds. Therefore, the acidic condition (during elution and liquid chromatography separation) disrupts the noncovalent bond connecting the light and heavy chain of the ADC. For trastuzumab emtansine and ADC2 DAR4, the reduction step is necessary to reduce the interchain disulfide bond, generating single antibody chain to improve chromatographic resolution. Optionally, deglycosylation canreduce the number of species, collapsing the different glycosylated forms together. Deglycosylation is generally suggested for more heterogeneous ADCs (i.e., ADC2 DAR4 and trastuzumab emtansine), where more varied DAR species contribute to the complexity of mass spectra. Because of the conjugation approach that employed all interchain disulfides, ADC2 DAR8 and ADC1 are more homogeneous, and glycosylation peaks coelute. Therefore, the deglycosylation of these ADCs was not necessary for quantification. Reduction and deglycosylation simplify the mass profile of the heterogeneous ADC and, thus, increase assay sensitivity. The processed ADC was then analyzed by LC-MS/MS, where payload-specific product ions dissociate from light and heavy chains. The integrated peak area of the signature payload product ions was then used for absolute quantification.

Impact of different MS1 isolation window resolution in the ZenoTOF 7600 system was also investigated. The charge-state distribution of light chain-1 payload (LC1) and heavy chain-3 payloads (HC3) of ADC1 is shown in Figure S1. 1800 *m*/*z* was chosen as a generic precursor ion with a wide isolation window to quantify both LC1 and HC3. The *m*/*z* peak closest to 1800 *m*/*z* is 1761.4 *m*/*z* (*z* = 14) for LC1 and 1792.7 *m*/*z* (*z* = 30) for HC3. Notably, both peaks are not the most abundant peaks in the charge distribution of the light or heavy chain. Nevertheless, we could dissociate the payload from both LC1 and HC3 with 1800 *m*/*z* as precursor ion and achieve adequate MS2 product peaks for quantification using open MS1 resolution (Figure S2, Figure S3A). With open resolution, more ions enter the collision cell compared to low resolution, which can result in MS2 saturation at higher concentration. This demonstrates the application of a more generic approach, which requires minimal method optimization for specific ADCs and can further simplify the workflow. If a more sensitive method is desired, using more abundant *m*/*z* for the precursor ion should be considered.

While the low MS1 resolution mode provides more targeted quantification, the open mode may be beneficial to quantify ADC with biotransformation. Common ADC biotransformations include hydrolysis, reduction/oxidation, cleavage by proteases, and linker-payload deconjugation.^8,11^ Biotransformation can occur on antibody, linker, and/or payload, which makes it challenging to quantify the biotransformed species. Multiple species with minor delta masses or of low abundance may impede accurate quantification by intact mode or at peptide level. In contrast, DF-MDMS quantification may overcome this issue by focusing on the payload signature ions only, by following these procedures: (1) set up a steep LC elution gradient for coelution of the parent and biotransformation species; (2) apply a slightly wider MS1 isolation window allowing both parent and biotransformed species to enter the collision cell (*m*/*z* range between parent and biotransformation species can be narrow at high charge state for >25k Da molecules such as payload conjugated to LC or HC); (3) quantify the payload signature product ions. ADC deconjugation is commonly observed for in vivo study samples. In the case of heterogeneous ADCs where DAR distribution can already be observed in reference material, absolute concentration of different species can be assessed with a calibration curve established for each DAR species. In the case of certain species information missing due to the sensitivity limitation, the average DAR information may be obtained by applying a steep LC gradient. Multiple species with various DARs can be merged into a single peak, thus allowing relative quantification of average DAR inherent to LCMS-based quantification methods; the capture affinity and ionization efficiency could be different between different DAR species.

**Figure 2:**
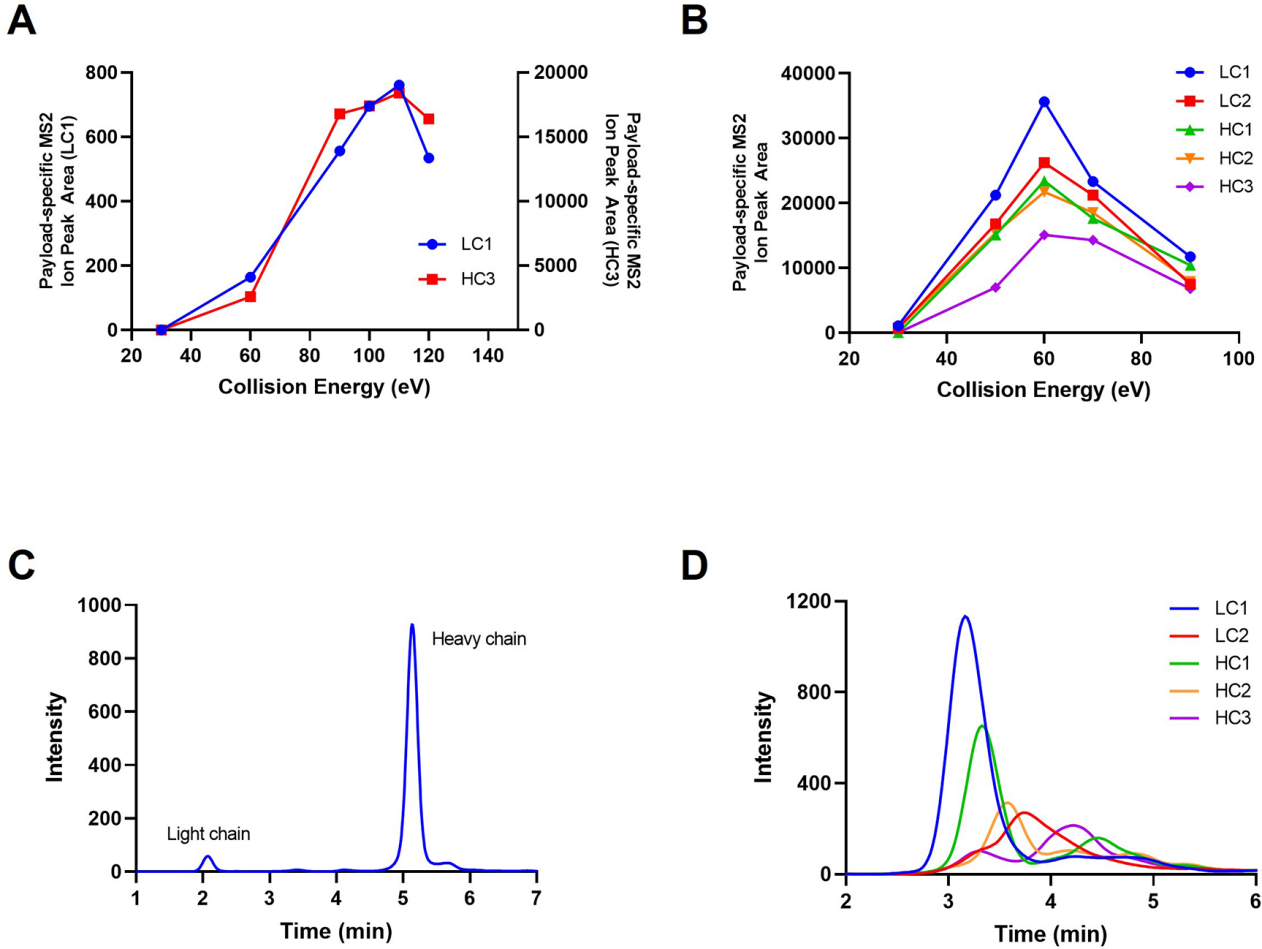
Collision energy optimization results of ADC1 and trastuzumab emtansine. CE optimization resulted in different peak areas of the payload specific MS2 ion for ADC1 (A) and trastuzumab emtansine (B). Representative MS2 XIC spectra are shown for ADC1 (C) and trastuzumab emtansine for different DAR species (D).

Different ADCs with various linker-payload structures require optimization of collision energy to generate maximized payload-specific product ion intensity for quantification. For each ADC, a series of experiments using the same parameters except for CE were performed. For ADC1, we observed that the signature payload MS2 ion intensity reached a plateau at around 100 eV and slightly decreased at 120 eV (Figure 2A), whereas for trastuzumab emtansine DM1-specific MS2 ion intensity reached its peak at 60 eV and then significantly dropped as CE continued to increase (Figure 2B). The product ion monitored for trastuzumab emtansine is potentially further fragmented at a higher CE. Hence, the CE was selected at 60 eV for trastuzumab emtansine.

Precursor or product ions can also be summed to enhance the sensitivity (Table S3). We can establish a calibration curve with *r* ≥ 0.985 and QC meeting acceptance criteria defined in ICH M10 by quantification with a single payload signature product ion.^12^ For some DAR species, summing precursor or product ions can increase sensitivity by lowering the LLOQ by 1.5- to 12-fold, improving the *r* value and QC passing rate. Additionally, peak integration parameters, such as MS2 XIC width (Figure S4), S/N integration threshold, Gaussian smooth width, and noise percentage, can be adjusted on a case-by-case basis for each ADC with different linker-payload and DAR distribution.

Thus, DF-MDMS quantification is a valuable tool for accurate DAR measurement of ADCs with noncleavable linker, such as trastuzumab emtansine. Figure 2D shows the payload specific MS2 XIC of various DAR species for light and heavy chains for trastuzumab emtansine. Compared to ADC1 MS^2^ XIC (Figure 2C), it shows the presence of multiple species (Figure 2D) with low intensity due to limited payload dissociation efficiency. Even with the above-mentioned challenges, the trastuzumab emtansine ADC concentration can be quantified using the DF-MDMS CID method with a linear dynamic range of 3000–90000 ng/mL for LC1 and 9000–90000 ng/mL for LC2 (light chain with 2 payloads). We were not able to establish a calibration curve for heavy chain species as the high heterogeneity of the conjugation site on the heavy chain in trastuzumab emtansine might play a role in the reduced ionization efficiency and consistency of heavy chain species.^10^ Optimization of the liquid chromatography strategy and alternative mass spectrometry methods should be considered for enabling absolute quantification of heterogeneous ADCs with different structures of payloads using DF-MDMS.

There are multiple factors that may contribute to the DF-MDMS assay sensitivity. While both DAR8 ADCs (ADC1 and ADC2) with homogeneous conjugation showed excellent sensitivity that can reach as low as 50 ng/mL, ADC2 DAR4 and trastuzumab emtansine had much higher LLOQ, even though ADC2 has the same payload as ADC1. ADCs with stochastic heterogeneous conjugation inherently have a distribution of DAR species on both light and heavy chains, thus affecting the LLOQ of each species. DAR can also affect LLOQ as moles of conjugated payload would impact signal intensity. Linker-payload intrinsic ionization and dissociation properties are also factors that can significantly affect the assay sensitivity. For TOP1i payload (AZ14170132), there are multiple signature product ions that can be used, whereas DM1 only has one unique MS2 product ion with low signal observed and is used for quantification (Figure S3). Therefore, the suitability of ADC quantification using the DF-MDMS CID approach should be considered in the context of linker-payload structure and conjugation approach.

In conclusion, we successfully established a novel digestion-free analytical method for absolute quantification of conjugated payloads from ADCs using CID-based mass spectrometry. Compared with other LCMS-based methods, this approach is cost and time effective, significantly reducing the workload related to enzymatic digestion and downstream data processing. It has been applied to multiple ADCs, including the noncleavable linker ADC trastuzumab emtansine. The results demonstrated that various types of ADCs can be quantified with reasonable sensitivity and straightforward data processing. We believe that this method can improve the throughput of bioanalysis, especially for noncleavable linker ADCs. It can also simplify analytical chemistry workflows for ADCs in samples with pure buffer, where no immunocapture and direct MS injection may be applied.

## Supporting Information Available

Charge-state distribution, calibration curves, payload signature product ions, XIC optimization data, tandem mass spectrometry, data processing and peak integration parameters, and quantification results of ADCs (PDF)
