# Supplementary material for: Digestion-Free Middle-Down Mass Spectrometry Method for Absolute Quantification of Conjugated Payload from Antibody-Drug Conjugates
Source: Anal Chem. 2024 Sep 19;96(42):16475–80. doi: 10.1021/acs.analchem.4c03383 (PMC11503511; doi:10.1021/acs.analchem.4c03383)
Supplement: Supplementary file 1 — ac4c03383_si_003.pdf [file ac4c03383_si_003.pdf]

Supporting Information

Digestion-Free Middle-Down Mass Spectrometry Method for Absolute Quantification of Conjugated Payload from Antibody-Drug Conjugates

Jiaqi Yuan[1,#], Hui Yin Tan[1,#], Yue Huang[1,*], Anton I. Rosenbaum[1,*]

[1]Integrated Bioanalysis, Clinical Pharmacology & Safety Sciences R&D, AstraZeneca, South San Francisco, CA 94080, USA

[#]J.Y. and H.Y.T contributed equally to this paper

*To whom correspondence should be addressed:

Anton I. Rosenbaum, Ph.D.

121 Oyster Point Blvd, South San Francisco, CA 94080

Email: anton.rosenbaum.phd@gmail.com

Yue Huang, Ph.D.

121 Oyster Point Blvd, South San Francisco, CA 94080

Email: yhuang@revmed.com

Table of Contents:

Figure S1. Charge state distribution of LC1 and HC3 of ADC1 S2

Figure S2. Calibration curves of ADCs S3

Figure S3. Payload signature product ions observed using CID S4

Figure S4. XIC optimization data processing results of ADC1 heavy chain at 50 ng/mL S5

Table S1. Tandem mass spectrometry (MS/MS) parameters on SCIEX 7600 Zeno TOF S6

Table S2. Data processing and peak integration parameters S7

Table S3. Quantification results of ADCs using different precursor and product ions S8

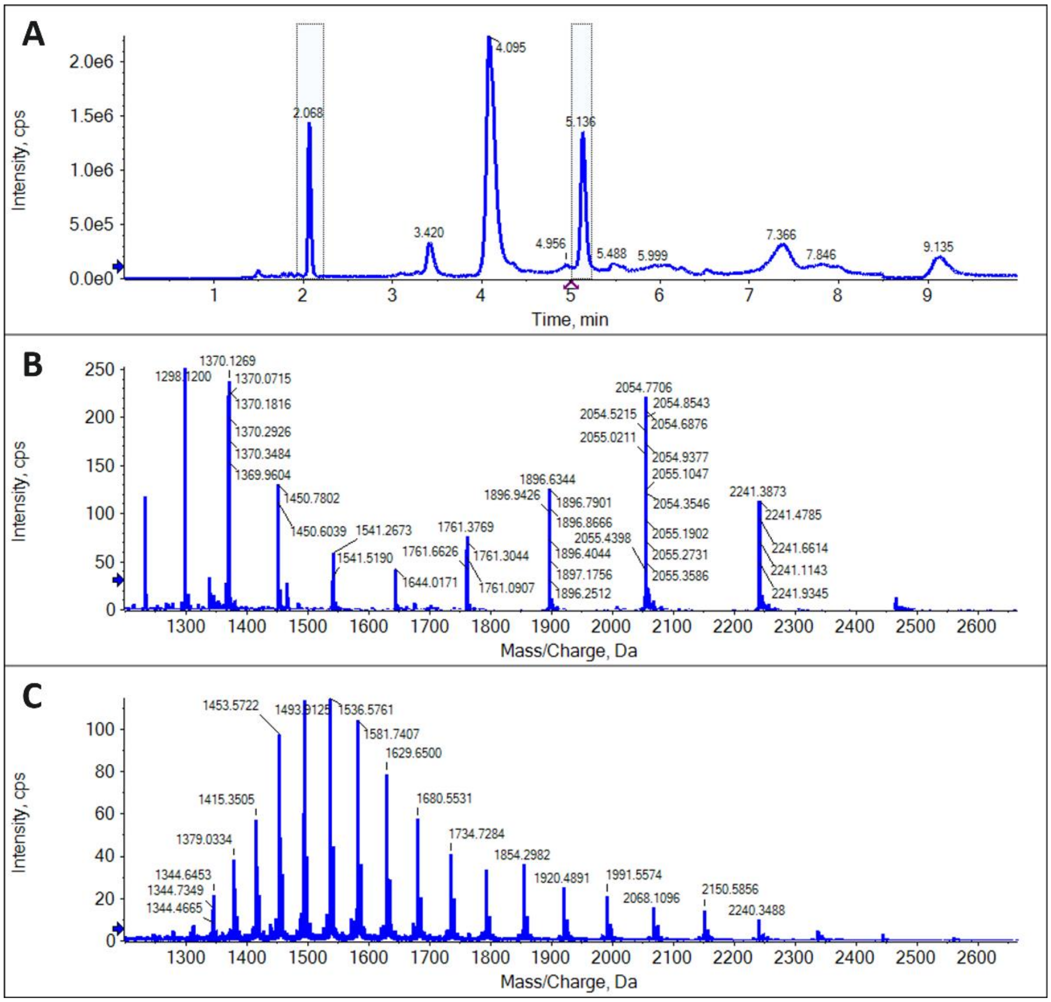

Figure S1. Charge state distribution of LC1 and HC3 of ADC1. (A) Total ion chromatogram of ADC1. (B) Charge state distribution of LC1 (retention time at 2.068 min). (C) Charge state distribution of HC3 (retention time at 5.136 min). Data was acquired from 1200-4000 m/z with spray voltage of 5500 V, de-clustering potential of 250 V, collision energy of 10 V.

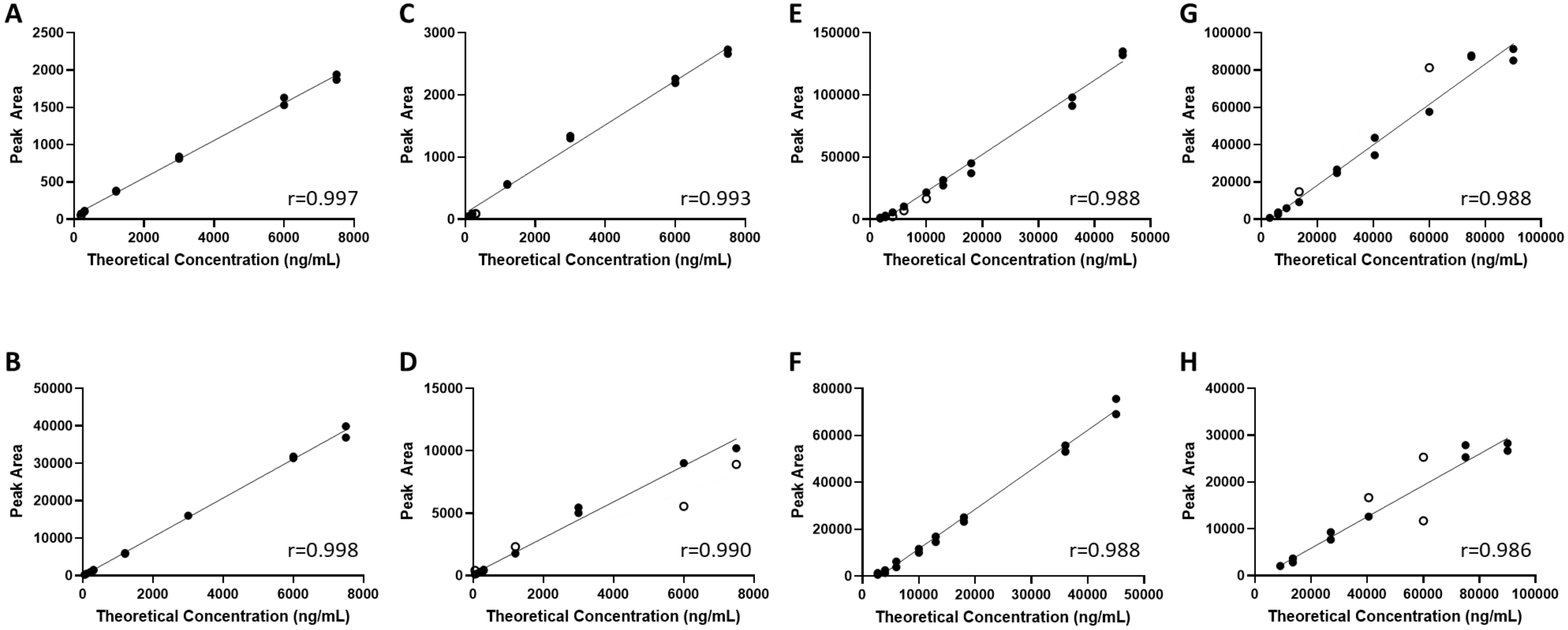

Figure S2. Calibration curves of ADCs: ADC1 LC1 (A) and HC3 (B), ADC2 DAR8 LC1 (C) and HC3 (D), ADC2 DAR4 LC1 (E) and HC2 (F), and trastuzumab emtansine LC1 (G) and LC2 (H). Filled circles represent standard replicates that meet acceptance criteria of ±20% / 25% (lowest standard level) for accuracy. Open circles represent standard replicates that fail to meet accuracy acceptance criteria. LC1-light chain with 1 payload, LC2-light chain with 2 payloads, HC2-heavy chain with 2 payloads, HC3-heavy chain with 3 payloads.

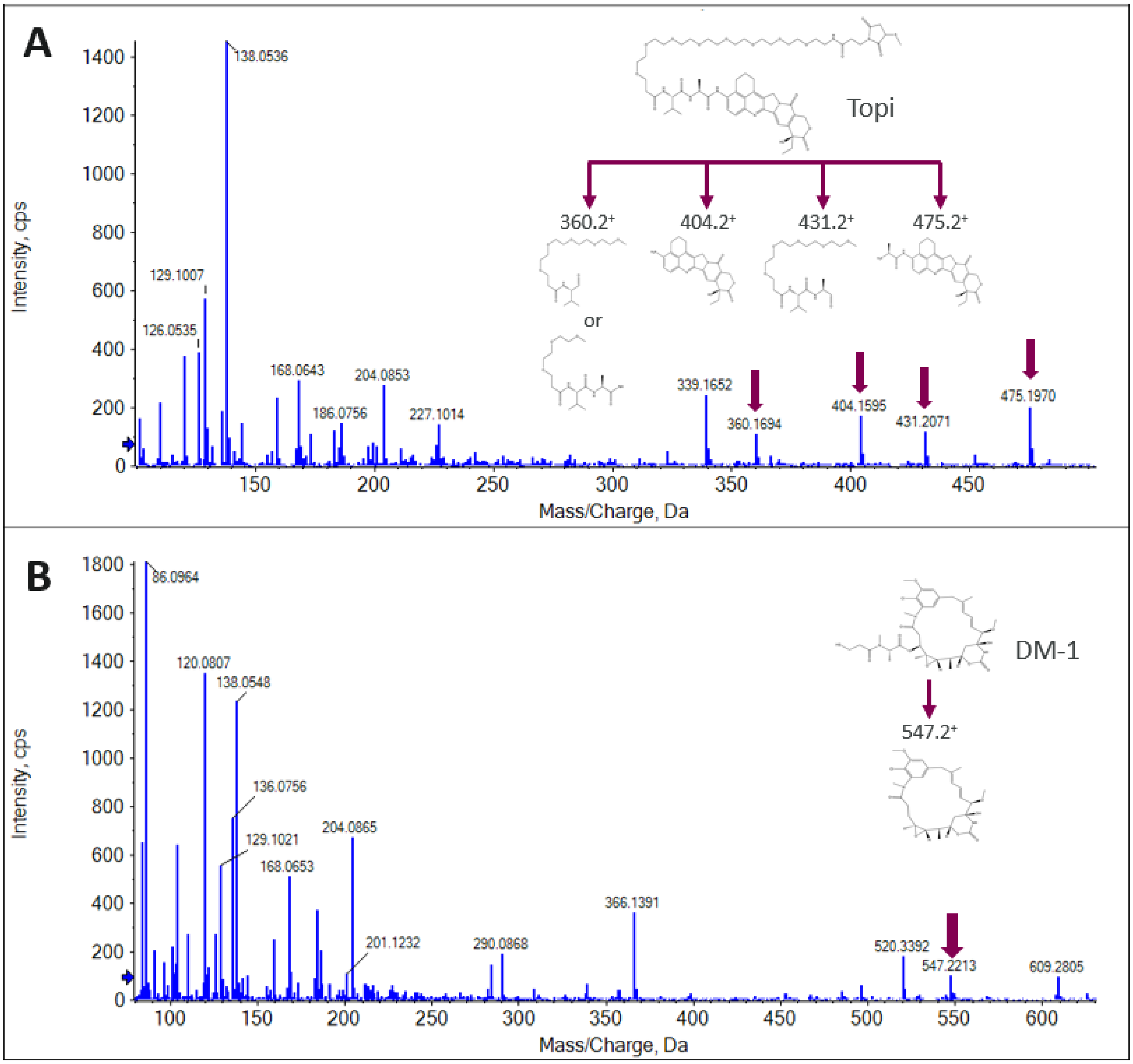

Figure S3. Payload signature product ions observed using CID. A) Signature product ion of TOP1i (AZ14170132) in ADC1. B) Signature product ion of DM-1 in trastuzumab emtansine.

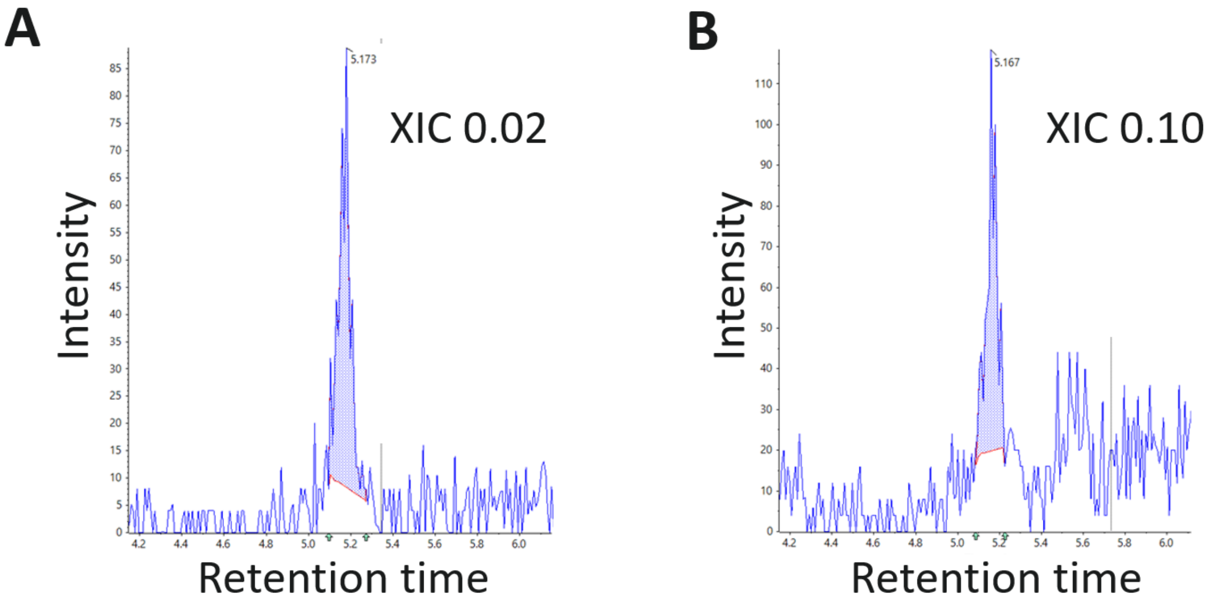

MS2 XIC width	Heavy chain standard curve r value	MQC area	S/N
0.01	0.995	~600	12
0.02	0.998	~1000	18
0.10	0.998	~1000	4

Figure S4. XIC optimization data processing results of ADC1 DAR8 heavy chain at 50 ng/mL. A) Spectrum of 4 ions (360.2^+, 404.2^+, 431.2^+, 475.2^+) summed with MS2 XIC width of 0.02. B) Spectrum of 4 ions (360.2^+, 404.2^+, 431.2^+, 475.2^+) summed with MS2 XIC width of 0.10. Standard curve r values and MQC area information are shown in the table. S/N = signal to noise ratio.

Table S1. Tandem mass spectrometry (MS/MS) parameters on Zeno TOF 7600.

Analyte	MS1					MS2				
	Accumulation time (ms)	Num. Time Bins to Sum	Range (m/z)	DP (V)	CE (V)	Accumulation time (ms)	Num. Time Bins to Sum	Range (m/z)	DP (V)	CE (V)
ADC1	250	6	1200-4000	250	10	250	8	100-2000	250	100
ADC2 (DAR 8)	250	6	1200-4000	250	10	250	8	100-2000	250	120
ADC2 (DAR 4)	250	6	500-2000	250	10	100	8	80-1600	250	120
Trastuzumab emtansine	250	6	500-2000	80	10	100	8	80-1600	80	60

DP = declustering potential, CE = collision energy

Table S2. MultiQuant data processing and peak integration parameters.

Analyte	Integration Algorithm	XIC width (Da)	S/N Integration Threshold	Smoothing/Gaussian Smooth Width (points)	Interference resolution/Noise Percentage (%)
ADC1	AutoPeak	0.02	2	NA	50
ADC2 (DAR 8)	AutoPeak	0.02	1	NA	95
ADC2 (DAR 4)	MQ4	0.05	0	6	90
Trastuzumab emtansine	MQ4	0.02	10	4	90

XIC = Extracted ion chromatogram, S/N = signal/noise ratio, NA = not applicable/not used in the integration algorithm

Table S3. Quantification results of ADCs using different precursor and product ions. Highlighted in green is the optimized assay for each DAR species of each ADC, highlighted in yellow is a passing assay, and highlighted in red is a failed assay.

LC = light chain, HC = heavy chain, r = Standard Curve correlation coefficient, STD = standard, QC = quality control

ADC1

Species	LC1					HC3				
Precursor Ion	1800	1800	1800	1800	1800	1800	1800	1800	1800	1800
Product Ion (z=1)	360.2	404.2	431.2	475.2	4 ions summed	360.2	404.2	431.2	475.2	4 ions summed
Calibration Range (ng/mL)	1200-7500	300-7500	1200-7500	300-7500	200-7500	50-7500	50-7500	50-7500	50-7500	50-7500
r	0.995	0.990	0.990	0.995	0.996	0.995	0.997	0.987	0.998	0.998
STD (Pass/Total)	7/8	7/10	8/8	9/10	11/12	16/16	15/16	13/16	15/16	16/16
QC (Pass/Total)	14/18	18/18	Fail	15/18	18/18	23/30	29/30	Fail	25/30	29/30

ADC2 DAR8

Species	LC1					HC3				
Precursor Ion	1800	1800	1800	1800	1800	1800	1800	1800	1800	1800
Product Ion (z=1)	360.2	404.2	431.2	475.2	4 ions summed	360.2	404.2	431.2	475.2	4 ions summed
Calibration Range (ng/mL)	1200-7500	1200-7500	1200-7500	1200-7500	100-7500	200-7500	200-7500	Fail	300-7500	50-7500
r	0.991	0.995	0.991	0.993	0.993	0.986	0.985	NA	0.988	0.990
STD (Pass/Total)	8/8	8/8	8/8	7/8	11/14	8/12	9/12	Fail	8/12	12/16
QC (Pass/Total)	15/20	Fail	10/12	18/20	16/20	Fail	11/16	NA	11/12	17/20

ADC2 DAR4

Species	LC1						HC1				
Precursor Ion	1375.3^{18+}	1375.3^{18+}	1375.3^{18+}	1375.3^{18+}	1375.3^{18+}	1375.3^{18+}	1416.7^{36+}	1416.7^{36+}	1416.7^{36+}	1416.7^{36+}	1416.7^{36+}
Product Ion (z=1)	360.2	404.2	431.2	475.2	4 ions summed	360.2, 431.2, 475.2	360.2	404.2	431.2	475.2	4 ions summed
Calibration Range (ng/mL)	1800-45000	Fail	1800-45000	1800-36000	1800-45000	1800-45000	Fail	Fail	Fail	Fail	Fail
r	0.989	0.981	0.988	0.988	0.989	0.987	0.912	0.925	0.898	0.916	0.982
STD (Pass/Total)	15/18	Fail	16/18	15/18	15/18	16/18	Fail	Fail	Fail	Fail	Fail
QC (Pass/Total)	24/36	NA	27/36	24/36	27/36	27/36	NA	NA	NA	NA	NA

Species	HC2						HC3				
Precursor Ion	1409.5^{37+}	1409.5^{37+}	1409.5^{37+}	1409.5^{37+}	1409.5^{37+}	1409.5^{37+}	1440.9^{37+}	1440.9^{37+}	1440.9^{37+}	1440.9^{37+}	1440.9^{37+}
Product Ion (z=1)	360.2	404.2	431.2	475.2	4 ions summed	360.2, 431.2, 475.2	360.2	404.2	431.2	475.2	4 ions summed
Calibration Range (ng/mL)	4000-45000	Fail	4000-45000	2700-45000	4000-45000	4000-45000	Fail	Fail	Fail	Fail	Fail
r	0.987	0.980	0.990	0.988	0.994	0.994	0.979	0.715	0.965	0.988	0.964
STD (Pass/Total)	14/14	Fail	13/14	14/14	14/14	14/14	Fail	Fail	Fail	Fail	Fail
QC (Pass/Total)	18/30	NA	25/30	26/30	25/30	25/30	NA	NA	NA	NA	NA

trastuzumab emtansine

Species	LC1			LC2		
Precursor Ion	1356^{18+}	1436^{17+}	2 ions summed	1335.3^{19+}	1409.6^{18+}	2 ions summed
Product Ion (z=1)	547.2	547.2	547.2	547.2	547.2	547.2
Calibration Range (ng/mL)	9000-90000	9000-90000	3000-90000	Fail	6000-90000	6000-90000
r	0.991	0.987	0.9881	0.981	0.986	0.988
STD (Pass/Total)	13/14	13/14	17/18	Fail	15/18	15/18
QC (Pass/Total)	23/24	20/24	26/30	NA	20/24	19/24

Species	HC1			HC2			HC3		
Precursor Ion	1364.7^{37+}	1402.5^{36+}	2 ions summed	1390.5^{37+}	1429.1^{36+}	Both	1455.7^{36+}	1497.3^{35+}	2 ions summed
Product Ion (z=1)	547.2	547.2	547.2	547.2	547.2	547.2	547.2	547.2	547.2
Calibration Range (ng/mL)	Fail	Fail	Fail	Fail	Fail	Fail	Fail	Fail	Fail
r	0.984	0.986	0.987	0.989	0.991	0.985	0.990	0.978	0.989
STD (Pass/Total)	Fail	Fail	Fail	Fail	Fail	Fail	Fail	Fail	Fail
QC (Pass/Total)	NA	NA	NA	NA	NA	NA	NA	NA	NA
